# Supplementary material for: WordCommentsAnalyzer: A windows software tool for qualitative research
Source: F1000Res. 2018 Sep 4;7:536. Originally published 2018 May 3. [Version 2] doi: 10.12688/f1000research.14819.2 (PMC6137411; doi:10.12688/f1000research.14819.2)
Supplement: Supplementary file 3 [file f1000research-7-17614-s0002.tgz › b2a79496-4ada-47c4-9682-a99ce867836d.zip!/Eight Iranian Health Journals/Archives of Rehabilitation_39.docx]

© Archives of Rehabilitation. This work is licensed under CC BY-NC 4.0 (https://creativecommons.org/licenses/by-nc/4.0/).

Volume & Issue: دوره 11 - شماره 1

Title: گزارش یک مورد کودک مبتلا به «هیدروسفالی و اوتیسم» و نتایج کاردرمانی در آن

http://rehabilitationj.uswr.ac.ir/article-1-442-fa.html

این تحقیق بصورت مطالعه موردی انجام گردید . در این پژوهش ، پسربچه 4 ساله ای که با تشخیص " هیدروسفالی و اوتیسم " توسط روانپزشک به یکی از کلینیک های کاردرمانی تهران ارجاع داده شده و تحت کاردرمانی قرار گرفت ، معرفی می گردد . همراهی علائم اوتیسم با هیدروسفالی سبب اختلال رشدی شدید در حیطه های ادراکی ، شناختی ، حرکتی ، ارتباطی و خودیاری شده بود . کاردرمانی برای این بیمار با هدف تسهیل ابعاد مختلف رشدی و ایجاد رفتارهای تطابق یافته ، به مدت یکسال انجام گرفت و در نتیجه ، اختلالات و مشکلات وی در اغلب ابعاد رشدی به میزان قابل توجهی کاهش یافت بعنوان نمونه ، تماس چشمی او افزایش چشمگیری یافت ، می توانست با خانواده و اطرافیان نزدیک ، تعامل نسبتا خوبی برقرار کند ، بیش فعالی ، خود آزاری و پرخاشگری او تا حد زیادی کاهش یافت ، می توانست نقاشی های ساده بکشد ، بدود و سه چرخه سواری کند . و با تداوم اقدامات توانبخشی ، در نهایت توانست در یکی از مدارس عادی دولتی تهران تحصیل در مقطع دبستان را آغاز نماید . آخرین پیگیری ها نشان می دهد کودک پایه پنجم دبستان را نیز با موفقیت ( با معدل 17 ) پشت سر گذاشته ، برای ورود به مقطع راهنمایی تحصیلی آماده می شود . نتایج این پژوهش ، توجه کاردرمانگران را به ارزیابی دقیق و همه جانبه کودکان مبتلا به هیدروسفالی ، ترکیب مناسب مداخلات درمانی ، همکاری نزدیک با سایر اعضای تیم توانبخشی و پیگیری مرتب وضعیت بیمار را حتی پس از ترخیص ، به همراه اهمیت مشارکت فعال والدین در درمان را مورد تاکید قرار می دهد .

Title: بررسی تأثیر طولانی مدت رویکرد درمانی بوبت بر روی یک کودک همی پلژی

http://rehabilitationj.uswr.ac.ir/article-1-440-fa.html

هدف: این تحقیق با هدف بررسی تأثیر طولانی مدت (6 سال ) رویکرد درمانی بوبت با استفاده از روشهای ابزاری نوین بر روی یک کودک همی پلژی صورت گرفت. روش بررسی: تحقیق حاضر یک مطالعه- تک موردی بوده و بر روی یک کودک مبتلا به فلج مغزی از نوع همی پلژی اسپاستیک انجام شد. برای ارزیابی وضعیت کیفی عملکرد حرکتی درشت از ارزیابی کلی بوبت و برای ارزیابی کمی، از ابزار ارزیابی عملکرد حرکتی درشت استفاده گردید. این ارزیابی ها به طور سالیانه و در مرداد ماه هر سال انجام شد. مداخلات درمانی، بر اساس تمرینات رویکرد درمانی بوبت به مدت 6 سال و متناسب با سطح توانائی کودک هفته ای یک بار انجام گردید. یافته ها: بر اساس سیستم طبقه بندی عملکرد حرکتی درشت، پس از دریافت مداخله، عملکرد حرکتی درشت مددجو ازسطح II به I ارتقا یافت. بر اساس ارزیابی کلی بوبت، در بدو ورود به کاردرمانی، مشکلات اولیه کودک شامل هایپرتونی، دامنه حرکتی محدود، فقدان حرکات مجزا بین اندامها، وجود الگوهای حرکتی غیر طبیعی در اندامها و هایپرلوردوز و اسکولیوز سمت چپ بدن بود. همچنین مهارتهای نشستن، غلتیدن از چپ به راست و خزیدن را انجام می داد. در سال ششم توانست مهارتهای راه رفتن، پریدن و دویدن سریع در محیط بیرون از منزل، لِی‌لِی کردن بر روی پای درگیر و بالا و پایین آمدن از پله به طور متناوب را به دست آورد. در ارزیابی با ابزار ارزیابی عملکرد حرکتی درشت، در اولین ارزیابی، نمره 09/46 و در سال ششم نمره 99/79 را کسب نمود. نتیجه گیری: برنامه های درمانی بوبت که بر اساس ارزیابی های دقیقی طرح ریزی شده باشند، می توانند از تثبیت الگوهای ناهنجار جلوگیری کرده و تا حد امکان توانایی‌های مددجو را به پایه رشدی طبیعی نزدیک نمایند.

Title: مقایسه تداخل دو تکلیف حافظه کوتاه مدت شنیداری و ثبات پاسچر بین افراد مبتلا به بی ثباتی عملکردی مچ پا و افراد سالم

http://rehabilitationj.uswr.ac.ir/article-1-439-fa.html

هدف: با توجه به اهمیت فرآیندهای آگاهانه از جمله فعالیت‌های شناختی در تنظیم و ثبات پاسچر و سازگاریهای احتمالی مطرح شده در سیستم عصبی مرکزی بیماران مبتلا به بی‌ثباتی عملکردی مچ پا، این تحقیق با هدف مقایسه الگوهای تداخل دو تکلیف حافظه کوتاه مدت شنیداری و ثبات پاسچر بین بیماران مبتلا به بی ثباتی عملکردی مچ پا و افراد سالم صورت گرفت. روش بررسی: در این مطالعه مقطعی و مورد– شاهدی، 15 بیمار مبتلا به بی ثباتی عملکردی مچ پا به روش ساده و در دسترس انتخاب و با 15 فرد سالم که با آنها جور شده بودند، با استفاده از الگوی تکلیف دوگانه، تحت بررسی و مقایسه قرار گرفتند. برای هر آزمودنی 6 مرحله ارزیابی، حاصل از دو سطح بی ثباتی دستگاه تعادل بایودکس و سه درجه دشواری تکلیف حافظه کوتاه مدت شنیداری انجام و در هر مرحله نمره عملکرد تکلیف پاسچر و تکلیف شناختی محاسبه شد. داده‌های حاصل با استفاده از آزمون آماری تحلیل واریانس برای اندازه های مکرر تجزیه وتحلیل شد. یافته ها: نتایج نشاندهنده تداخل بین تکلیف پاسچر و تکلیف حافظه کوتاه مدت شنیداری در بیماران مبتلا به بی ثباتی عملکردی مچ پا و افرادسالم بود (۰/۰۵>P)، ولی تداخل در دو گروه اختلاف معناداری نداشت. نتیجه‌گیری: یافته‌های حاصل، اختلال در روند تجزیه و تحلیل اطلاعات را در ضایعه ای عضلانی– اسکلتی– محیطی همچون بی‌ثباتی عملکردی مچ پا تأیید کرد. به‌کارگیری برنامه های تمرین درمانی جهت تسهیل روند تجزیه و تحلیل اطلاعات در روند توانبخشی این بیماران و استفاده از الگوهای پیش رونده تکلیف دو گانه، با درجات دشواری متفاوت تکلیف شناختی، مؤثر به نظر می‌رسد.

Title: الگوی مصرف مواد در معتادان گمنام

http://rehabilitationj.uswr.ac.ir/article-1-437-fa.html

هدف: شناسائی الگوی مصرف مواد در جامعه و همه گیر شناسی آن از مهمترین اقدامات لازم جهت ارزیابی، پیگیری و کنترل مصرف این مواد محسوب می گردد. هدف پژوهش حاضر بررسی الگوی سوء مصرف داروها و روشهای مصرف آنها در گروه معتادان گمنام می باشد. روش بررسی: در این مطالعه توصیفی– تحلیلی، به روش تمام شمار، 300 نفر از معتادان مراجعه کننده به یک مرکز درمانی معتادان گمنام شهر تهران (که به صورت در دسترس انتخاب شده بود) شرکت کرده و اطلاعات لازم شامل مشخصات دموگرافیک افراد، وضعیت اجتماعی و اقتصادی آنان و نوع و نحوه مصرف مواد، با استفاده از پرسشنامه محقق ساخته از آنان اخذ و داده‌های حاصل با استفاده از آزمون مجذور خی مورد بررسی قرار گرفت. یافته ها: ۸۰ درصد از جمعیت معتادان گمنام را مردان و حدود %2/65 از این جمعیت را افراد دارای سنین کمتر از 40 سال تشکیل می‌دادند. بیش از دو سوم افراد مورد مطالعه دارای مشاغل آزاد بوده و 2/21 درصد از آنان بیکار بودند. %32 از بیماران واجد تحصیلات دانشگاهی و شایعترین مواد مصرفی در افراد مورد مطالعه به ترتیب شامل تریاک (88%)، سیگار (%7/76) و الکل (%63) بوده و %34 افراد مواد را از طریق تزریق مصرف می نمودند. مصرف الکل (P=0/033)، حشیش (P<0/0001)، کوکائین (P=0.009) و اکستازی (P<0.001) به طور معناداری در گروه کمتر از 40 سال بیشتر از افراد مسن تر بود. نتیجه گیری: به نظر می رسد که روند مصرف مواد در قشر جوان و تحصیل کرده در حال افزایش است. الگوی مصرف در جوانان بیشتر به سمت مواد روانگردان از قبیل کوکائین، حشیش و اکستازی می باشد.

Title: تأثیر خدمات کاردرمانی روانی – اجتماعی بر کیفیت زندگی بیماران مبتلا به اختلالات روانی مزمن

http://rehabilitationj.uswr.ac.ir/article-1-436-fa.html

هدف: ارتقاءء کیفیت زندگی هدفی مهم از مراقبتهای بهداشتی در روانپزشکی شناخته می شود. در تحقیق حاضر با انجام مداخلات کاردرمانی روانی- اجتماعی بر روی بیماران دچار اختلالات روانی مزمن، به بررسی کیفیت زندگی آنها پرداخته شد. روش بررسی: این مطالعه مداخله ای به صورت شبه تجربی می باشد و بر روی 24 بیمار مبتلا به اختلالات روانی مزمن مراجعه کننده به مرکز روزانه سینا به عنوان گروه مداخله و 50 بیمار دارای اختلالات روانی مزمن که خدمات کاردرمانی دریافت نمی کردند به عنوان گروه مقایسه انجام شد. نمونه ها از طریق نمونه گیری ساده و در دسترس انتخاب شدند. ابزار پژوهش «پرسشنامه کیفیت زندگی مُراجع ویسکانسین» بود که شامل ۸ خرده مقیاس می‌باشد. خدمات کاردرمانی که شامل گروه درمانی، فعالیت درمانی و هنردرمانی بود، طبق برنامه مرکز هفته ای 2 بار، به مدت ۹۰ دقیقه و به طول 3 ماه بر روی گروه مداخله انجام شد. در این مدت هر دو گروه تحت نظر روانپزشک و دارودرمانی قرار داشتند. برای تجزیه و تحلیل آماری از آزمون‌های تی مستقل و تی زوجی استفاده شد. یافته ها: قبل از مداخله در هیچ یک از حیطه ها و کیفیت زندگی کل بین دو گروه تفاوت معناداری وجود نداشت، ولی بعد از مداخله میانگین نمره رضایت از زندگی، فعالیت اشتغالی، سلامت و آسایش روانی، سلامت جسمانی و کیفیت زندگی کل (۰/۰۰۱>P) و همچنین روابط اجتماعی (۰/۰۰۵=P) و وضعیت مالی (۰/۰۰۳=P) دو گروه تفاوت معناداری داشت. میانگین نمره علائم (۰/۲۲۷=P) و فعالیت روزمره زندگی (۰/۰۲۰=P) دو گروه بعد از مداخله نیز تفاوت معناداری نشان نداد. نتیجه گیری: خدمات کاردرمانی روانی- اجتماعی بر میزان رضایتمندی و کیفیت زندگی بیماران اختلالات روانی مزمن مؤثر است.

Title: ویژگیهای روان سنجی نسخه فارسی مقیاس ترس از حرکت تمپا در بیماران ایرانی مبتلا به کمردرد

http://rehabilitationj.uswr.ac.ir/article-1-435-fa.html

هدف: ترس از درد و حرکت یکی از عوامل مهم ایجاد ناتوانی عملکردی در بیماران مبتلا به کمردرد است که بسیاری از محققین آن را حتی از خود درد ناتوان کننده‌تر می‌دانند. هدف تحقیق حاضر بررسی خصوسیات روان سنجی نسخه فارسی مقیاس ترس از حرکت تمپا که به عنوان یک ابزار رایج در دنیا برای سنجش میزان ترس از حرکت و صدمه در بیماران کمردردی به کار می‌رود بود. روش بررسی: در این مطالعه توصیفی و متدولوژیک که از نوع اعتبارسنجی و ابزارسازی می‌باشد، 232 نفر بیمار مبتلا به کمردرد مراجعه کننده به برخی کلینیکها و بیمارستانهای در دسترس شهر تهران با نمونه گیری ساده انتخاب شده و مورد ارزیابی قرار گرفته و 61 نفر از آنها برای آزمون مجدد جهت سنجش تکرارپذیری شرکت کردند. جهت اعتبارسنجی خصوصیات روان سنجی نسخه ایرانی مقیاس ترس از حرکت تمپا همخوانی درونی، همبستگی آیتم با نمره کل، تکرارپذیری آزمون ـ بازآزمون و اعتبار سازه مورد ارزیابی قرار گرفت. ابزارها و آزمون‌های به کار رفته علاوه بر مقیاس ترس از حرکت تمپا، آزمون آنالوگ دیداری برای سنجش شدت درد، آزمون باورهای منجر به ترس ـ اجتناب جهت سنجش ترس از درد در فعالیتهای بدنی و شغل و آزمون ناتوانی رولند ـ موریس جهت سنجش ناتوانی بالینی بودند. روش‌های آماری شامل محاسبه ضریب‌های آلفای کرونباخ، همبستگی پیرسون و همبستگی درون گروهی (ICC) و آزمون‌های کولموگروف– اسمیرنوف و تی زوجی بود. یافته ها: همخوانی درونی مقیاس خوب و ضریب آلفای کرونباخ برای 17 آیتم آن معادل 796/0 بود. همبستگی آیتم ـ نمره کل برای هر آیتم محاسبه گردید که به غیر از چهار آیتم، سایر موارد همبستگی مناسبی را نشان دادند. ضریب همبستگی درون گروهی در ارزیابی تکرارپذیری 86/0 به دست آمد. جهت محاسبه اعتبار سازه، ضریب همبستگی پیرسون بین مقیاس ترس از حرکت تمپا با دو خرده مقیاس فعالیت بدنی و شغل آزمون باورهای منجر به ترس ـ اجتناب به ترتیب 59/0 و 32/0 و با آزمون ناتوانی رولند ـ موریس 60/0 به دست آمد. نتیجه گیری: نسخه فارسی مقیاس ترس از حرکت تمپا دارای روایی و پایایی لازم جهت سنجش ترس از حرکت/صدمه در بیماران کمردردی ایرانی می باشد و قابل استفاده در کلینیک ها و مراکز درمانی و یا جهت اهداف تحقیقی است.

Title: رابطه گرایش های مذهبی و ابعاد شخصیت با رضایت دانشجویان کارورز توانبخشی از تجارب بالینی

http://rehabilitationj.uswr.ac.ir/article-1-434-fa.html

هدف: هدف از پژوهش حاضر ارزیابی رابطه گرایش های مذهبی و ابعاد شخصیت با رضایت دانشجویان کارورز توانبخشی از تجارب بالینی خود به عنوان یک درمانگر بود. روش بررسی: دراین مطالعه توصیفی، 318 دانشجوی کارورز توانبخشی شامل 140 نفر دانشجوی فیزیوتراپی، 97 نفر دانشجوی کاردرمانی و 81 نفر دانشجوی گفتاردرمانی به صورت نمونه گیری طبقه ای-تصادفی انتخاب شدند. داده ها با استفاده از پرسشنامه های مذهب گرایی اسلامی، پنج عامل شخصیت و رضایت از تجربیات بالینی جمع آوری و با استفاده از محاسبه ضریب همبستگی پیرسون و تحلیل رگرسیون چندگانه گام به گام تحلیل شد. یافته‌ها: نتایج نشان داد که در تمام دانشجویان کارورز توانبخشی، بین رضایت از تجارب بالینی با مذهب گرایی، ارزنده سازی مذهبی و سه بعد شخصیت برون گرایی، توافق و وجدانی بودن همبستگی مثبت معنادار و با سازمان نایافتگی مذهبی و روان رنجور خویی همبستگی منفی معنادار دارد(001/۰>P). نتایج تحلیل رگرسیون برای پیش بینی رضایت از تجربیات بالینی در دانشجویان نشان داد که در دانشجویان فیزیوتراپی متغیرهای برون گرایی، مذهب گرایی و توافق، در دانشجویان کاردرمانی متغیرهای برون گرایی، وجدانی بودن و کامجویی و در دانشجویان گفتاردرمانی متغیرهای مذهب گرایی و سازمان نایافتگی مذهبی وارد معادله رگرسیون شدند. نتیجه‌گیری: براساس یافته های پژوهش می توان نتیجه گرفت که گرایش های مذهبی و ابعاد شخصیت قادر به پیش بینی رضایت از تجربیات بالینی در دانشجویان فیزیوتراپی، کاردرمانی و گفتاردرمانی هستند.

Title: استانداردسازی سیاهه ابراز خشم صفت ـ حالت 2 اسپیلبرگر

http://rehabilitationj.uswr.ac.ir/article-1-441-fa.html

هدف: باتوجه به دارا بودن قابلیت بالای پژوهشی و درمانی سیاهه ابراز خشم صفت ـ حالت 2 اسپیلبرگر، پژوهش حاضر با هدف بررسی ویژگی های روان‏سنجی و هنجاریابی آن در جمعیت دانشجویان ایرانی انجام یافت. روش بررسی: روش تحقیق توصیفی– پیمایشی از نوع ابزار‌سازی است. گروه نمونه مورد مطالعه 1140 نفر از دانشجویان دختر و پسر دانشگاه تهران (با میانگین سنی 92/21 و انحراف معیار 89/2) بود. از این تعداد 554 نفر دختر (6/48 درصد) و 586 نفر پسر (40/51 درصد) بودند که 1080 نفر آنها مجرد (7/94 درصد) بود. این دانشجویان به شیوه خوشه‌ای چند مرحله‌ای انتخاب و از آنها خواسته شد به دو ابزار پاسخ دهند. یکی از این دو ابزار سیاهه ابراز خشم صفت_ حالت 2 اسپیلبرگر و دیگری یکی از ابزارهای مقیاس خشم چند بعدی، مقیاس خصومت مهار شده افراطی، پرسشنامه شادکامی آکسفورد، سیاهه‌ی هوش هیجانی و سیاهه شخصیتی نئو بود. یافته‌ها: دختران و پسران در اکثر خرده مقیاسهای خشم صفت– حالت تفاوتی ندارند. ضرایب همبستگی بین اغلب خرده مقیاسهای خشم صفت– حالت و همچنین ضرایب همبستگی بین خرده مقیاسهای خشم صفت– حالت با مقیاسهای موازی معنادار است (P>0/05). همجنین میانگین کلیه خرده مقیاسهای خشم صفت– حالت در دو گروه دارای هوش هیجانی بالا و پایین تفاوت معنادار نشان دادند (P>0/05). تحلیل عاملی نمایانگر وجود عوامل چندگانه همسان با فرم اصلی در هر یک از بخش‌های سیاهه خشم صفت– حالت است. برای هر یک از خرده‌مقیاسهای خشم حالت– صفت جداول هنجاری براساس نمرات T استخراج شد. نتیجه گیری: سیاهه ابراز خشم صفت– حالت 2 اسپیلبرگر از روایی و اعتبار مناسبی جهت سنجش خشم در جوانان ایرانی برخوردار است. لذا از این سیاهه می توان به عنوان ابزاری جهت سنجش خشم صفت و حالت در پژوهش ها و مجموعه های بالینی استفاده نمود.
